# Hemodynamic control during cardiopulmonary bypass and the incidence of postoperative delirium– a post hoc analysis

**DOI:** 10.1186/s12871-025-03141-8

**Published:** 2025-05-26

**Authors:** Helena Claesson-Lingehall, Birgitta Olofsson, Yngve Gustafson, Alexander Wahba, Micael Appelblad, Staffan Svenmarker

**Affiliations:** 1 Department of Nursing, Umeå University, Umeå, SE 901 87 Sweden; 2 Department of Community Medicine and Rehabilitation, Geriatric Medicine, Umeå University, Umeå, Sweden; 3 Heart Centre, Department of Public Health and Clinical Medicine, Umeå University, Umeå, Sweden; 4 Norwegian University of Circulation and Medical Imagining, Trondheim, Norway; 5 Faculty of Medicine and Health, Örebro University, Örebro, Sweden

**Keywords:** Delirium, Postoperative delirium, Neurological complication, Postoperative cognitive complications, Cardiac surgery, Cardiopulmonary bypass

## Abstract

**Background:**

Delirium is a common neurological complication after cardiac surgery. The purpose of the present study was to analyze the association between hemodynamic fluctuations during cardiopulmonary bypass (CPB) and the incidence of postoperative delirium (POD) in patients undergoing cardiac surgery with CPB.

**Methods:**

This post hoc analysis included one-hundred-ninety-five (*n* = 195) patients aged ≥ 65 years of whom seventy (*n* = 70) patients developed POD. Intraoperative hemodynamic variables specifically related to the conduct of CPB were digitally recorded at 1-minute intervals. Variables outside the presumed safe boundaries for mean arterial pressure (MAP), systemic perfusion flow index– L/min/BSA (QBSA_I_), systemic venous oxygen saturation (S_V_O_2_) and arterial oxygen delivery– ml/min/BSA (DO_2_) were defined and analyzed with reference to indices of area under the curve (AUC) and the relative proportion of registrations related to POD. POD was diagnosed according to DSM-5 criteria based on a test battery performed preoperatively and repeated twice postoperatively. Statistical tests used to verify observations outside the predefined norm included the Mann-Whitney U test and the chi-squared test.

**Results:**

Markers of hemodynamic control during CPB showed significant associations with POD. Both DO_2_ (*P* = 0.02) and QBSA_I_ (*P* < 0.001) identified POD patients outside the predefined upper and lower safety limits. S_V_O_2_ values > 84% (*P* < 0.001) werealso associated with the development of POD. The number of S_V_O_2_ registrations below the lower safety limit was negligible, why statistical analysis seemed not useful. No association between MAP and POD registrations was identified.

**Conclusions:**

This study revealed a clear association between markers of hemodynamic control and POD. These associations were most pronounced for DO_2_ and QBSA_I_. The detected association between high S_V_O_2_ and POD warrants further insight.

## Introduction

Neurological complications after cardiac surgery with cardiopulmonary bypass (CPB) are still very common. These injuries are classified as Type-I injuries, which involve structural brain damage, such as stroke, whereas Type-II injuries refer to more subtle functional impairments characterized by fluctuations in cognition and attention, manifested as postoperative delirium (POD) [1]. The reported incidence following cardiac surgery ranges from 14 to 54% [2–5]. Delirium is a multifactorial condition, resulting from a complex interplay of pre-existing and precipitating risk factors [4, 6]. Delirium usually occurs within the first 3 to 7 days after surgery and it is strongly associated with increased morbidity and mortality, postoperative cognitive decline and dementia [7–10].

During cardiac surgery with CPB, systemic perfusion flow and blood pressure are typically maintained within specific targets to ensure adequate organ perfusion [11]. Despite advancements in the intraoperative management, CPB still contributes to the development of POD. Several risk factors have been identified including the duration of CPB [2, 12], high positive fluid balance, prolonged mixed venous oxygen desaturation [13] and systemic perfusion rates below estimated targets [2].

The present study aims to test the hypothesis that hemodynamic fluctuations during CPB are associated with POD in patients ≥ 65 years undergoing cardiac surgery.

## Materials and methods

### Study population

The following study refers to a post hoc analysis of a previous publication [14] from our group registered in The European Union Drug Regulating Authorities Clinical Trials Database (EudraCT) (018-002385-39). The present protocol was approved by the Swedish Ethical Review Authority (Dnr 2023-02241-02). The study population comprised 195 elective patients aged ≥ 65 years who were scheduled for routine cardiac surgery via cardiopulmonary bypass (CPB) at the Heart Centre, Umeå University, Sweden. Seventy patients (36%) developed postoperative delirium.

### Main objective

The objective of this study was to identify associations between hemodynamic control during CPB and the development of POD. The use of high-resolution data recordings of both blood pressure and systemic perfusion flow enables a sensitive statistical analysis.

### Assessments of postoperative delirium

The diagnosis of POD was set according to the DSM-5 criteria [15] via the results from: the Mini Mental State Examination Second Edition Standard Version (MMSE-2 SV) [16], The Organic Brain Syndrome Scale (OBS) [17], the Nursing Delirium Screening Scale (Nu-DESC) [18], The Richmond Agitation Sedation Scale (RASS) [19] and the Glasgow Coma Scale (GCS) [20]. The assessments for delirium were coordinated with other included measurements starting preoperatively and repeated after extubating on days 1(+ 1) and day 3 (+/-1) postoperatively. Extended information is provided in our reference publication [14].

### Intraoperative procedures

Surgery was performed according to standard procedures for coronary artery bypass grafting and valve replacements, isolated or combined. In general, central anastomoses were sutured behind a side-biting clamp. The left internal mammary artery was in most cases attached to the anterior descending branch of the left coronary artery. Cardioplegic arrest was accomplished by cold St Thomas II 1:4 blood cardioplegia, both of which were administered at 10^◦^C.

Patient monitoring included radial artery and central venous blood pressure, 5-lead electrocardiography and transcutaneous oxygen saturation. General anesthesia combined with propofol, fentanyl, rocuronium and sevoflurane. Patients were ventilated to normocapnia. Systemic blood pressure was controlled by norepinephrine and phenylephrine, whereas cardiac function was improved the combined use of epinephrine, milrinone or levosimendan.

Roller pumps and membrane oxygenation were used for CPB aimed to preserve a mean arterial pressure (MAP) > 50 mmHg and the mixed venous saturation (S_v_O_2_) > 75% via systemic perfusion flow adjustments and vasoactive drugs. The target body temperature was 34 °C. Shed blood was retransfused continuously. Anticoagulation from administered heparin aimed to maintain the activated clotting time above 480 s. The targets for arterial carbon dioxide and the oxygen concentration (5 kPa / 15 kPa) were verified via intermittent blood gas analyses.

### Data collection

Data specifically related to the conduct of CPB was digitally registered once a minute and stored in a local hospital database (PDMS Metavision by iMD Soft, Tel Aviv, Israel). The hematocrit value obtained in the registry was converted to a hemoglobin value [21].

### Definition of hemodynamic indices

**Fig. 1:**
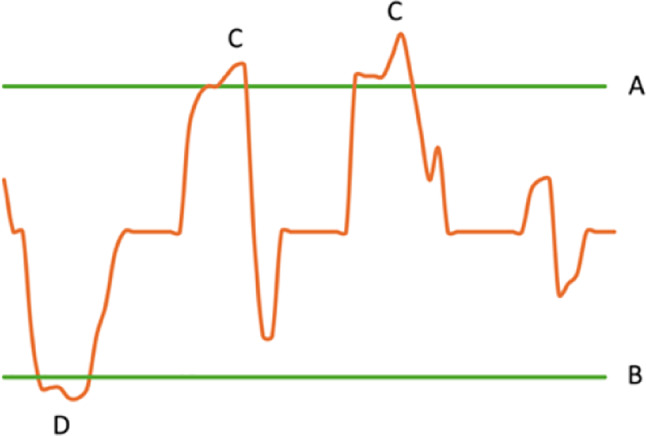
Schematic description of references used to calculate deviations outside a predefined upper (**A**) and lower (**B**) limit. Episodes above (**C**) and below (**D**) are classified and presented as time, area under the curve (AUC) and the area under the curve per minute (mAUC). This model was implemented to present systemic venous oxygen saturation, arterial blood pressure, body surface area as a systemic perfusion flow marker and the arterial oxygen delivery as guidance for cardiopulmonary bypass perfusion flow control

A priori definitions of hemodynamic indices with corresponding upper and lower limits used to control systemic perfusion flow and mean arterial pressure are presented in Fig. 1. These indices are arbitrary defined or in correspondence with existing guidelines [11].

1 $$QBSA_{I} = Systemic\:Perfusion\:Flow (Liter/min)/BSA (m^{2})$$

2 $$O_{2}\:Content\:(ml/L) = Hemoglobin\:(g/L) \star 1.34$$

3 $$DO_{2} = O_{2}\: Content \star QBSA_{I}$$

The definitions of arbitrarily safe boundaries used to test established indices of systemic perfusion flow control during cardiopulmonary bypass are defined in Table 1. Reference for the perfusion flow was set by the S_v_O_2_ value and maintained above 75% [14].

**Table 1:** Classification of tested safe boundaries of systemic perfusion flow control during cardiopulmonary bypass with reference to the development of postoperative delirium

Classification	Definition	Lower limit	Upper limit
Systemic venous oxygen saturation (%)	70 ±20%	> 56	< 84
Mean arterial pressure (mmHg)	60 ±30%	> 42	< 78
Systemic perfusion flow index (QBSA_I_)	2.4 ±10%	> 2.16	< 2.64
Arterial oxygen delivery (ml/min (DO_2_)	270 ±15%	> 232	< 312

Definition of abbreviations and equations: BSA = Body surface area (m^2^), QBSA_I_ = L/min/BSA, O_2_ Content (ml/L) = Hemoglobin (g/L) * 1.34, DO_2_ = O2 Content * QBSA_I_

### Statistical methods

The collected data were plotted in histograms and QQ-plots to establish distribution patterns combined with the Shapiro-Wilk test. Normally distributed data were analyzed via Student’s t-test; otherwise, the Mann-Whitney U-test was used. Categorical data were tabulated and associations were verified by the chi square test. Central tendency was presented as the mean or the median value combined with the standard deviation and the interquartile range or as the minimum and maximum values as indicated. A two-sided P-value < 0.05 was regarded as statistically significant. Statistical analyses were performed in Microsoft^®^ Excel version 16.93 and IBM^®^ SPSS Statistics version 29.

## Results

### Pre and intraoperative characteristics

Patients diagnosed with POD were older (Table 2). Other predisposing possible risk factors included the duration of surgery, cardiopulmonary bypass and aorta occlusion in addition to higher positive fluid balance and urine output (Table 3).

### Kernel density plot presenting the hemodynamic variables and postoperative delirium

**Fig. 2:**
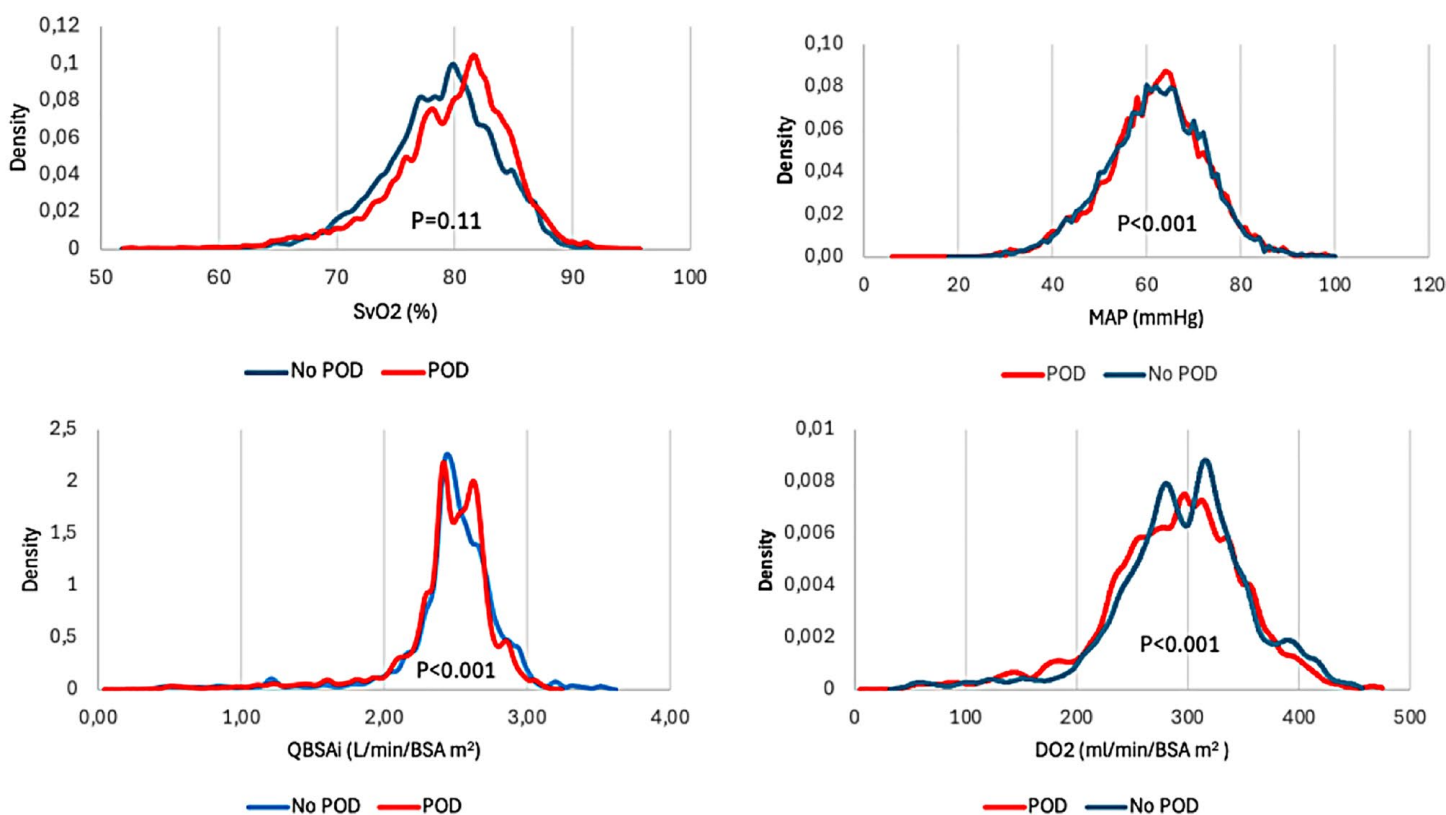
Kernel density plots showing systemic venous oxygen saturation (S_v_O_2_), mean arterial pressure (MAP), systemic perfusion flow index (QBSA_I_) and measured arterial oxygen delivery (DO_2_) during cardiopulmonary bypass in patients with and without postoperative delirium (POD) registered once a minute, including > 17 000 registrations per parameter

The presentation provides an overview of the density representation of each hemodynamic variable investigated in this study comparing patients with and without POD. Overall, there were significant differences in the mean arterial pressure (MAP), systemic perfusion flow index (QBSA_i_) and the level of arterial oxygen delivery (DO_2_), however, except for the systemic venous oxygen saturation (S_v_O_2_) as summarized in Fig. 2.

**Table 2:** Baseline patient information

	All	POD	No POD	*P*-value
Age (yrs)	73.1 ±4	74.7 ±4	72.2 ±4	< 0.001
Female sex (%)	27.2	28.6	26.4	0.74
Body mass index (kg/m^2^)	27.4 ±4	27.2 ±4	27.5 ±4	0.65
EuroSCORE II	1.89 (1.9)	2.30 (2.4)	1.59 (1.3)	0.005
University education (%)	21.5	14.3	25.6	0.07
Hypertension (%)	81.5	84.3	80.0	0.46
Diabetes type 2 (%)	22.1	22.9	21.6	0.84
Type of surgery (%)				0.26
Coronary artery bypass	39.5	40.8	38.1	
Aortic valve replacement	39.5	42.9	36.1	
Aortic valve and Coronary bypass	16.4	11.2	21.6	
Other	4.6	5.1	4.1	
Ventricular function (%)				0.71
Mildly reduced	17.4	21.4	15.2	
Moderately reduced	11.3	11.4	11.2	
Severely reduced	2.1	1.4	2.4	
Atrial fibrillation (%)	19.5	25.7	16.0	0.10
Pulmonary obstructive disease (%)	3.6	1.6	7.1	0.046
Use of CPAP (%)	10.3	14.3	8.0	0.17
Hemoglobin (g/L)	135 ±14	135 ±14	135±13	0.89
NT-proBNP (ng/L)	445 (965)	538 (1326)	335 (856)	0.07
CRP HS (mg/L)	1.4 (3)	2.6 (3.7)	1.2 (2.5)	0.03
Platelets (10^9^/L)	225 (68)	208 (68)	234 (72)	0.01

Results are presented as means ±SD or medians (IQR). CPAP = continuous positive airway pressure. CRP HS = High sensitivity C-reactive protein. NT-proBNP = N-terminal pro-B-Type natriuretic peptide. CRP = C-reactive protein

**Table 3:** Intra and postoperative results

	All	POD	No POD	*P*-value
Surgery (min)	177 (60)	193 (63)	167 (56)	< 0.001
Cardiopulmonary bypass (min)	89 (42)	99 (45)	85 (39)	< 0.001
Aorta occlusion (min)	62 (35)	71 (41)	56 (30)	0.002
Fluid balance (mL)	1724 (856)	1891 (1052)	1678(797)	0.04
Urine output (mL)	420 (390)	510 (386)	370 (375)	0.002
Propofol (mg)	272 (151)	282 (186)	264 (139)	0.22
Fentanyl (µg)	800 (150)	775 (225)	800 (175)	0.24
	*Postoperative results*			
Chain drain volume 24 h (ml)	640 (430)	700 (603)	580 (380)	0.02
Red cell transfusion (units)*	0 (2)	2 (2)	0 (1)	< 0.001

The results are presented as medians (IQR). * Including intraoperative transfusion

### Area under the curve representing deviations outside a predefined normal range

Both S_v_O_2_ and variance were significantly greater in the POD group, whereas the duration and magnitude of the AUC for S_v_O_2_ below 56% and above 84% during CPB differed insignificantly (*P* > 0.05). No significant differences were detected for MAP, either for the overall level or variance or for measures of the AUC below 42 mmHg and above 78 mmHg. The calculated QBSA_i_ marker of systemic perfusion flow was somewhat lower in the POD group 2.44 [0.2] versus 2.45 [0.2] (*P* < 0.001). AUC values for the QBSA_i_ below 2.16 and above 2.64 were not significantly different. A DO_2_ level less than 232 ml/min was clearly associated with the development of POD. The AUC was 408 (DO_2_*min) compared to 231 (DO_2_*min) for patients in the POD group (*P* < 0.008). The DO_2_ variance was also significantly greater among POD patients (*P* < 0.001). AUC for DO_2_ above 312 ml/min levels showed no statistically significant intergroup differences. Summary in Table 4.

### An overview of different markers of systemic perfusion flow control during cardiopulmonary bypass and the incidence of postoperative delirium

#### Systemic venous oxygen saturation and postoperative delirium

**Fig. 3:**
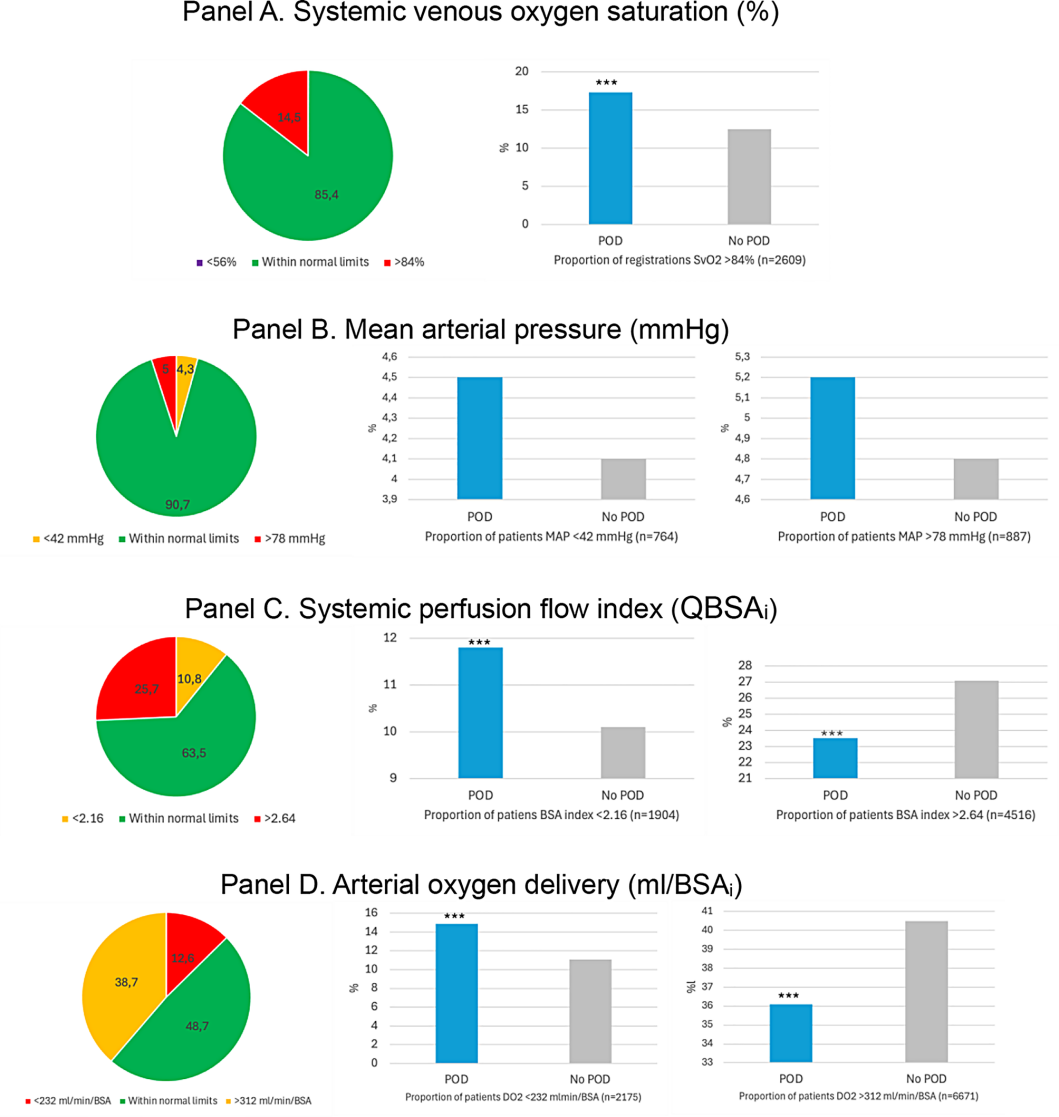
Classification of systemic venous oxygen saturation (%) in panel **A**, mean arterial blood pressure (mmHg) in panel **B**, systemic perfusion flow index (L/min/BSA_i_) in panel **C** and arterial oxygen delivery (ml/min/BSA_i_) in panel **D**. Registrations within and outside a predefined normal range (depicted to the left). The bar charts show the proportion of registrations in patients diagnosed with and without postoperative delirium (POD). *P* < 0.001 (***). Note that central venous oxygen saturation < 56% lacks enough registrations to be visualized in the pie chart of panel **A**

More than 85% of the registered S_v_O_2_ levels were within the predefined normal upper and lower limits (56–84%). Maintaining the S_v_O_2_ level above the 84% marker was associated with a significantly higher incidence of POD based on the bases of proportional analyses of registered values in the database. The lack of registrations below the 56% S_v_O_2_ threshold disabled further statistical analysis. Figure 3– Panel A.

#### Mean arterial pressure and postoperative delirium

More than 90% of the registered MAP notations were within the predefined normal range (42–78 mmHg). No associations were identified between different blood pressure levels and the postoperative incidence of POD. Figure 3– Panel B.

#### Systemic perfusion flow index QBSA_i_ and postoperative delirium

More than 60% of the QBSA_i_ registrations were within predefined limits (2.16–2.64). A threshold below QBSA_i_ <2.4 was associated with a higher incidence of POD. While QBSA_i_ in assess of 2.8 was not associated with POD. Figure 3– Panel C.

#### Arterial oxygen delivery and postoperative delirium

The proportion of DO_2_ registrations within normal predefined limits (232–312 ml/min/BSA) was 48.7%. DO_2_ levels less than 300 ml/min/BSA were associated with a higher POD incidence in contrast to levels > 300 ml/min/BSA Fig. 3– Panel D.

**Table 4:** Markers of hemodynamic control during cardiopulmonary bypass in patients with and without postoperative delirium

	POD	No POD	*P*-value
**Central Venous oxygen saturation (S**_**v**_ **O**_**2**_ **)**			
S_v_O_2_ (%)	80.2 [5.2]	78.9 [4.7]	< 0.001
S_v_O_2_ variance	26.5	23.1	< 0.001
S_v_O_2_ <56%			
Time (min)	1.0 (1–2)	1.0 (1–2)	0.61
AUC (S_v_O_2_*min)	54 (52–105)	54 (52–110)	0.44
mAUC (AUC/Time)	52.7 (52–54)	53.7 (52–55)	0.36
S_v_O_2_ >84%			
Time (min)	3.0 (1–72)	2.0 (1–79)	0.06
AUC (S_v_O_2_*min)	254 (84-6239)	170 (84-6716)	0.11
mAUC (AUC/Time)	85.1 (84–96)	85.1 (84–94)	0.91
**Mean arterial pressure (MAP)**			
MAP (mmHg)	62 [6]	62 [7]	0.41
MAP variance	113.9	112.9	0.52
MAP < 42 mmHg			
Time (min)	1.0 (1–13)	1.0 (1–13)	0.49
AUC (MAP*min)	41 (6-491)	41 (26–493)	0.65
mAUC (AUC/Time)	38 (6–41)	38 (26–41)	0.76
MAP > 78 mmHg			
Time (min)	2.0 (1–10)	2.0 (1–16)	0.12
AUC (MAP*min)	170 (79–820)	160 (79-1344)	0.07
mAUC (AUC/Time)	82 (79–94)	82 (79–100)	0.15
**Systemic perfusion flow index (L/BSA m** ^**2**^ **)**			
QBSA_i_ (L/min/BSA m^2^)	2.44 [0.2]	2.45 [0.2]	< 0.001
QBSA_i_ variance	0.13	0.15	0.49
Systemic perfusion flow index < 2.16 (L/min/BSA m^2^)			
Time (min)	2.0 (1–30)	2.0 (1–14)	0.29
AUC (QBSA_i_ *min)	2.1 (0.5–46)	1.9 (0.3–21)	0.18
mAUC (AUC/Time)	1.3 (0.4–1.9)	1.2 (0.3–1.9)	0.13
Systemic perfusion flow index > 2.64 (L/min/BSA m^2^)			
Time (min)	3.0 (1–30)	4 (1–94)	0.70
AUC (QBSA_I_ *min)	8.9 (2.9–87)	11.6 (2.9–283)	0.73
mAUC (AUC/Time)	3.0 (2.9–3.2)	3.0 (2.9–3.4)	0.71
**Arterial oxygen delivery DO**_**2**_			
DO_2_ (ml/min/BSA m^2^)	288 [58]	292 [48]	0.20
DO_2_ variance	3979	3721	< 0.001
Arterial oxygen delivery < 232 (ml/min/BSA m^2^)			
Time (min)	2.0 (1–50)	2.0 (1–28)	0.10
AUC (DO_2_*min)	408 (55-9863)	231 (32-11802)	0.008
mAUC (AUC/Time)	195 (44–232)	176 (32–232)	0.002
Arterial oxygen delivery > 312 (ml/min/ BSA m^2^)			
Time (min)	3.0 (1-142)	3.0 (1-124)	0.82
AUC (DO_2_*min)	944 (312-51179)	942 (312-46623)	0.73
mAUC (AUC/Time)	324 (312–447)	324 (312–449)	0.96

Episodes outside predefined limits were based on the duration (time) of the total area under the curve (AUC) and the mean area under the curve (mAUC). Data represent the median accompanied by the interquartile range [] or the minimum and maximum values (). The variance is equal to the statistical notification. POD = postoperative delirium. S_v_O_2_ = Systemic venous oxygen saturation. MAP = Mean arterial pressure. QBSA_I_ = Systemic perfusion flow index. DO_2_ = Arterial oxygen delivery

## Discussion

The aim of this study was to investigate whether the POD incidence is associated with hemodynamic control of the mean arterial pressure, systemic perfusion flow index, central venous oxygen saturation and arterial oxygen delivery during CPB in patients ≥ 65 years of age undergoing cardiac surgery. Our main findings indicate that circulatory control during CPB is significantly associated with the development of POD, why its role as a possible risk factor should be considered.

The observed incidence of POD was 36% which is well within the range reported in the literature [2–5]. POD was assessed prospectively pre- and postoperatively using a range of tests, as part of a prospective trial as outlined previously [14]. The focus was on the acute development of POD and not on long-term aspects. Thus, no evaluation beyond 3 days postoperatively was performed.

### Influence of arterial oxygen delivery (DO_2_)

Several risk factors for POD have been suggested in the literature [4, 6]. The role of hemodynamic control during CPB is of specific interest from this perspective. A large meta-analysis revealed that low DO_2_ was correlated with POD and stroke in 8 out of 10 studies [22]. However, the quality of the evidence was not high and only a subgroup of studies confirmed a significant difference in DO_2_ between groups [22]. Moreover, outcomes of interest, such as time below a certain DO_2_ threshold and the AUC are not universally employed and thresholds vary [22]. In this study it was attempted to alleviate some of the shortcomings of previous publications by using a range rather than a threshold to measure time and AUC. The calculation of the range was based on a threshold for DO_2_ commonly referred to in guidelines. A 15% margin was added to define the upper and lower limits of the range. Our results confirm that low DO_2_ values are associated with the development of POD in patients over 65 years of age. POD was significantly more common in patients where DO_2_ below the lower margin was registered and less common in those with a DO_2_ above the upper margin. We also found that the AUC below the lower margin of normal DO_2_ was significantly greater than in patients who didn’t develop POD. However, the perfusion time below the range was short and not significantly different between groups. Overall, there seems to be a clear effect of DO_2_ on POD development. However, differences between groups were not large, except for the AUC in patients with a submarginal DO_2_.

### Influence of mean arterial pressure

We were unable to find a significant association between MAP and POD. Notably, only 10% of the recordings were outside of the range, 3.4% below the lower threshold of the predefined range (42-78mmHg). This finding likely reflects that abnormal blood pressure values were well tailored by the operating team.

Previous studies have shown inconsistent results when comparing high versus low MAP targets during CPB, with no difference in mortality or cognitive function [23], whereas others have reported fewer neurological complications in high-MAP perfusion [24]. A recent systematic review and meta-analysis assessed morbidity and mortality associated with high versus low blood pressure targets during CPB in a total of 1116 patients [25]. The main findings indicated no differences in clinical outcomes, such as delirium, cognitive decline, stroke, AKI or mortality, whereas a small number of trials indicated that high blood pressure may increase the risk for blood transfusion.

Novel methods recommend that the MAP should be optimized in accordance with the autoregulatory mechanisms of the cerebral blood flow [26]. This is possible by matching MAP with real-time measurements of the cerebral blood flow using either near infrared oximetry [26] or transcranial Doppler [27]. Brown and colleagues reported a 45% reduction in POD incidence with the implementation of this method [27]. The introduction of bedside monitoring opens a new window for blood pressure control during CPB [26].

### Influence of systemic perfusion flow

In our study systemic perfusion flow was guided by the S_v_O_2_ level, with a lower limit of 75%, however an upper limit was not set. The underlying theory behind involves balancing oxygen delivery with oxygen demand [28–31]. The response from the vascular system was measured as the mean arterial blood pressure, which frequently requires pharmacological adjustment.

S_v_O_2_ recordings exceeding the 84% upper margin were more common among POD patients. Kernel density analysis revealed a clear cutoff at 80% when S_v_O_2_ registrations started systematically to overlap. Fewer than 15% of data points were outside of the upper predefined range. The corresponding AUC and time analyses indicated the same results; however, the results did not reach statistical significance. The finding that hyperoxemia is associated with POD may seem unlikely [32] based on findings from Lopez et al. However, in an earlier report from the same author, hyperoxic cerebral reperfusion was revealed to increase the risk of POD by 65% [33]. These opposing results suggest that hyperoxemia is only a risk factor for POD in conjunction with episodes of cerebral hypoxia or circulatory disturbances. Since cerebral oximetry monitoring was not included as part the study protocol, we can only speculate whether high S_v_O_2_ levels explain the development of POD in this study.

The findings for S_V_O_2_ may seem counterintuitive, since one would expect S_V_O_2_ to be positively linked to oxygen delivery. However, the correlation between S_V_O_2_ and DO_2_ was only 9% (not reported), which explains why this assumption was not fulfilled. A low S_V_O_2_ was previously reported to increase the incidence of POD [4]. In this study, we were unable to confirm this association, since virtually no S_V_O_2_ datapoints were below the lower margin.

There was a significant difference in the body surface-indexed perfusion flow between the groups, but the numerical difference was not clinically relevant. The perfusion flow index was outside of the predefined frame in 36.5% of the measurements. The incidence of POD was significantly increased in patients with flow values below the lower margin and significantly reduced in those with values above the upper margin. However, time and AUC for values outside of the range were not significantly different between patients with and without POD.

### Limitations

This is a post hoc investigation employing material that was prospectively collected for another project [14]. The selected margins for the normal range of the tested parameters were based on current guidelines and a group discussion. Naturally, the definition is arbitrary to some degree, which is why the results should be interpreted while keeping this in mind. Background characteristics differed and most notably for patient age and EuroSCORE in addition to several other possible confounders. Degree of statistical power differed significantly between the tests employed in this study.

## Conclusion

This study identified several potential strategies for reducing the risk of POD following cardiac surgery, including the optimization of oxygen delivery, and central venous oxygen saturation. Effective hemodynamic management during CPB may mitigate the risk of POD. To confirm this relationship and gain a comprehensive understanding of the underlying mechanisms contributing to POD, as well as its broader clinical implications, further investigation is warranted.

## Data Availability

The ethical application we submitted did not include information about making the data available to others. Unfortunately, this means that making the data visible to others outside the research group is not ethically justifiable.

## Abbreviations

AUC: Area under the curve
CPB: Cardiopulmonary bypass
DO_2_: Arterial oxygen delivery
MAP: Mean arterial pressure
POD: Postoperative delirium
QBSA_i_: Systemic perfusion flow index
S_v_O_2_: Systemic venous oxygenation saturation
